# Timing of high-intensity pulses for myocardial cavitation-enabled therapy

**DOI:** 10.1186/2050-5736-2-20

**Published:** 2014-10-02

**Authors:** Douglas L Miller, Chunyan Dou, Gabe E Owens, Oliver D Kripfgans

**Affiliations:** 1 Department of Radiology, University of Michigan Health System, 3240A Medical Sciences Building I, 1301 Catherine Street, Ann Arbor 48109-5667, USA; 2 Department of Pediatrics, University of Michigan Health System, Ann Arbor, MI, USA

**Keywords:** Myocardial contrast echocardiography, Cavitation microlesions, Arrhythmia, Cardiac myocyte necrosis, Hypertrophic cardiomyopathy

## Abstract

**Background:**

High-intensity ultrasound pulses intermittently triggered from an ECG signal can interact with circulating contrast agent microbubbles to produce myocardial cavitation microlesions of potential therapeutic value. In this study, the timing of therapy pulses relative to the ECG R wave was investigated to identify the optimal time point for tissue reduction therapy with regard to both the physiological cardiac response and microlesion production.

**Methods:**

Rats were anesthetized, prepared for ultrasound, placed in a heated water bath, and treated with 1.5 MHz focused ultrasound pulses targeted to the left ventricular myocardium with an 8 MHz imaging transducer. Initially, the rats were treated for 1 min at each of six different time points in the ECG while monitoring blood pressure responses to assess cardiac functional effects. Next, groups of rats were treated at three different time points: end diastole, end systole, and mid-diastole to assess the impact of timing on microlesion creation. These rats were pretreated with Evans blue injections and were allowed to recover for 1 day until hearts were harvested for scoring of injured cardiomyocytes.

**Results:**

The initial results showed a wide range of cardiac premature complexes in the ECG, which corresponded with blood pressure pulses for ultrasound pulses triggered during diastole. However, the microlesion experiment did not reveal any statistically significant variations in cardiomyocyte injury.

**Conclusion:**

The end of systole (R + RR/3) was identified as an optimal trigger time point which produced identifiable ECG complexes and substantial cardiomyocyte injury but minimal cardiac functional disruption during treatment.

## Background

Myocardial contrast echocardiography provides the capability of perfusion imaging with ultrasound [1,2]. Contrast agents, which consist of suspensions of stabilized microbubbles, are injected intravenously and increase the echogenicity of the myocardium. When relatively high pressure-amplitude ultrasound pulses are used, for example, greater than about 1 MPa at 2 MHz [3], the microbubbles are destroyed, clearing the contrast enhancement and revealing the relative microbubble content by comparing the before and after images. If the process is repeated with different intervals between the clearance pulses, then the relative perfusion of different regions is discernable. In addition to the diagnostic application, this imaging method can be enhanced for therapeutic purposes. The strong, cavitation-like response of the microbubbles at relatively high peak rarefactional pressure amplitudes (PRPAs) can lead to a variety of microscale bioeffects in contrast-enhanced diagnostic ultrasound [4]. The local bioeffects, or microlesions, are attributable to nucleation of inertial cavitation [5,6]. The bioeffects have the potential to be harnessed in several therapeutic strategies [7].

Myocardial contrast echocardiography has been shown to be capable of causing lethal injury of cardiomyocytes, which leads to scattered microlesions throughout the scanned region [8]. The microlesions, which consist of one or a few lethally injured cardiomyocytes, heal within a few weeks with minimal scarring [9]. Recently, this phenomenon was proposed as a means of ultrasound therapy in which the scattered microlesions would innocuously heal, leaving a measured reduction in tissue volume [10]. This noninvasive and relatively gentle method of tissue reduction might be advantageous, for example, in treatment of hypertrophic cardiomyopathy (HCM) and other ventricular hypertrophies. Echocardiography is the optimum method for diagnosis of these conditions, and extension of the imaging methods to therapeutic application may be particularly advantageous clinically. The treatment would be repeatable and avoid methods with relatively high impact and patient risk such as alcohol ablation [11,12] or surgical myectomy [12,13]. Parameters for myocardial cavitation-enabled therapy (MCET) were optimized for microlesion production using 1.5-MHz-focused ultrasound pulses, which were trigged at mid-cardiac cycle and guided by 8-MHz diagnostic ultrasound [10]. Evans blue-stained cardiomyocyte scores (SCSs) were used to assess the relative efficacy with 2 or 4 MPa pulses, 1:4 or 1:8 trigger intervals, and 5 or 10 cycle pulses. The results indicated a major increase in efficacy by increasing the PRPA from 2 MPa, in the diagnostic range, to 4 MPa, which often exceeded the microlesioning level needed for therapeutic tissue reduction. The increased intervals and pulse durations did not yield significant increases in the effects. These findings suggested that MCET can potentially become a clinically robust therapeutic tool after refinement and control of the treatment process.

The purpose of this study was to examine the impact of this method on cardiac function during treatment and to determine the best time point in the ECG for application of the therapeutic pulses. We hypothesized that ultrasound-induced arrhythmia, blood pressure perturbation, and microlesion production would have significant dependences on the timing of the therapy pulses within the heart cycle. In addition, for treatment in many patients, significant perturbation of cardiac function might pose significant risks and become an important consideration for treatment planning. An optimum combination of minimal cardiac functional disruption and treatment efficacy is needed for this therapy method. The lethal injury of cardiomyocytes is accompanied by premature complexes (PCs) in the ECG [14], but the meaning of these complexes was not fully understood. In order to establish whether or not these signals announce actual premature ventricular contractions, or possibly only pulseless electrical activity, the heart function was investigated using real-time blood pressure monitoring for a 4-MPa treatment at six different time points in the cardiac cycle. In addition, to determine if cardiomyocyte microlesion formation is dependent on cardiac cycle and presumed myocardial blood vessel volume (systole = reduced blood volume; diastole = increased volume), treatment efficacy evaluated by microlesion formation was compared with pulses delivered at end diastole, end systole, or mid-diastole. The results provided key information for treatment planning with regard to the response of the heart during treatment and the therapeutic outcome.

## Materials and methods

### Animal preparation

All *in vivo* animal procedures were conducted with the approval and guidance of the University Committee on Use and Care of Animals. Twenty-eight male Sprague-Dawley rats (Charles River, Wilmington, MA, USA) weighing 331 ± 33 g were tested for this study. One rat was used for preliminary setup and one did not survive for the 1-day examination. Rats are the most common mammalian animal model for cardiovascular research, particularly for studies requiring numerous animals and test groups [15]. Rat models for hypertrophic cardiomyopathy are available [16], but often difficult to obtain. However, even Sprague-Dawley rats have a tendency toward left ventricular hypertrophy [17] and therefore seem to be a good model for this study.

Rats were anesthetized by intraperitoneal (IP) injection of a mixture of ketamine (90 mg/kg) and xylazine (9 mg/kg), and the left thorax was shaved and depilated for ultrasound transmission. A 24-gauge cannula was inserted into a tail vein for IV injections of contrast agent. In seven rats, a catheter with a pressure sensor (model SPR-320 2 F catheter, Millar Instruments, Houston, TX, USA) was inserted into a femoral artery and the sensor advanced to the abdominal aorta. The blood pressure signal was amplified with a bridge amplifier and calibrated using a calibration kit (FE221 and MLA1052, ADInstuments, Inc., Colorado Springs, Co, USA). In 21 rats, Evans blue dye in saline (20 mg/ml) was injected IV at a dose of 100 mg/kg as a vital stain for cardiomyocytes [8]. ECG electrodes were applied and the rats were positioned in a warmed water bath for ultrasound scanning and treatment. The ECG and blood pressure signals were digitized (Powerlab 4/30, ADInstruments Inc., Colorado Springs, CO, USA), recorded, and analyzed with the aid of software (Chart Pro 5, v. 5.5.5, ADInstruments Inc., Colorado Springs, CO, USA).

### Ultrasound

Ultrasound exposure was provided by a laboratory system with guidance by diagnostic ultrasound imaging. The 1.9-cm-diameter therapy transducer with a 3.8-cm focus was mounted on a moveable gantry together with the 8-MHz diagnostic probe used at 8 MHz, 5-cm depth, and low -14-dB power setting (mechanical index 0.2). The setup provided targeting of the therapy beam and for low-power imaging of the heart during exposure. The aim was adjusted to focus the beam into the window between ribs and between the sternum and lung and to enter the heart approximately at the middle of the left ventricle. The exposure consisted of bursts of eight pulses triggered from the ECG signal using optimal parameters of five cycle pulses with 4-MPa PRPA, determined previously [10]. For this study, the pulse amplitudes were re-measured in the water bath using a calibrated hydrophone (Model 805, Sonora Medical systems Inc., Longmont, Co, USA). The pulses were triggered 1:4 at a desired time point relative to the heart cycle determined by the ECG signal.

The ECG signal and the blood pressure signal, when monitored, were recorded for 1 min before treatment, during treatment, and for 1 min after treatment. The ECG signal provides a crude monitor of the treatment because premature complexes in the ECG at the trigger points are strongly correlated with lethal cardiomyocyte injury. For rats with overnight recovery, the ECG also was recorded for 1 min prior to euthanasia. Definity® (Lantheus Medical Imaging, Inc., North Billerica, MA, USA) ultrasound contrast agent was infused at 5 μl/kg/min, diluted 100:1 in sterile saline. After placement and stabilization of a rat in the water bath for a few minutes, the R wave-to-R wave (RR) interval was measured and the desired trigger point set. For exposure, the probes were set, the Definity infusion was initiated, and the therapy exposure was started at the appearance of the contrast agent in the left ventricle.

### Cardiomyocyte scoring

The development of microlesions was assessed in samples obtained 1 day after treatment. The microlesions were scored on the basis of Evans blue staining [10]. Each heart was evaluated from up to 40 frozen sections cut at 200-μm spacing, and the scores were totaled in an effort to characterize the overall impact. For these therapy experiments, the large accumulations of stained cells in frozen sections were often difficult to accurately count and the resulting scores remained qualitative (rather than an actual cell count). As an additional measure of the overall cardiomyocyte injury, plasma samples were collected after 1 day and analyzed for troponin I with an ELISA assay kit (Rat Cardiac Tn-I (Ultra Sensitive), Life Diagnostics Inc., West Chester, PA, USA). This cardiac enzyme is a sensitive indicator of cardiac injury in rodents [18] and provides an indicator of the total amount of cell killing.

### Experimental plan

The study was conducted in two parts. The first part tested six rats using varying trigger timing with blood pressure monitoring. The RR interval of each rat was determined, and the trigger time point was stepped by increments of RR/6 for six 1-min treatments. In order to average any trend with time for the six treatments, the sequence was started at a different time point for each rat. Specifically, the first through sixth rat was started at the R wave (R + 0RR/6), the R wave plus a delay of RR/6 (R + RR/6), R + 2RR/6, R + 3RR/6, R + 4RR/6, and R + 5RR/6, respectively. Rats in part 1 had the ECG and blood pressure signals evaluated and samples of the treated area of the heart taken for histology approximately 15 min after the conclusion of treatments. The second part of the study involved 20 rats in four groups treated as shams or treated with pulse triggering at end diastole (R wave), at end systole (R + 2RR/6), or at mid-diastole (R + 4RR/6). These rats had Evans blue injection and evaluation of the microlesion production in hearts sampled the next day.

Results are reported as the means plus or minus one standard deviation or plotted with standard error bars. The data were evaluated by statistical analysis software (SigmaPlot for Windows v. 11.0, Systat Software Inc., San Jose, CA, USA). Student’s *t*-tests were used to compare means of the measured parameters, with statistical significance assumed at *P* < 0.05. In addition, repeated-measures ANOVA analysis was used to examine the significance of results for part 1, in which each rat had all six test conditions.

## Results

### Part 1 results

**Figure 1:**
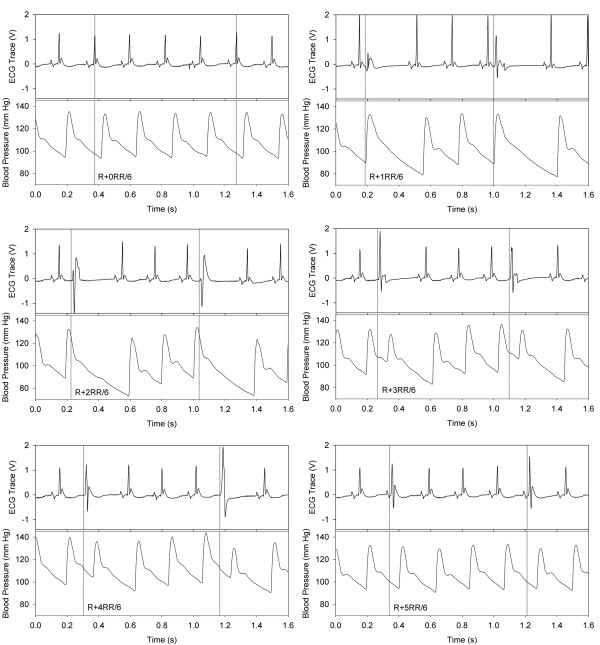
**Comparison plots of the ECG and blood pressure signals for one rat in part 1.** The timing of the triggers for the high-intensity therapy pulses was incremented by RR/6 and is shown by the vertical dotted lines. The treatment pulses typically elicited a premature complex in the ECG, except for triggering at the R wave (R + 0RR/6). The blood pressure signal shows the heart functional response, with premature blood pulses generated for R + 3RR/6 and later trigger points.

In part 1, the six rats had an average RR interval of 230 ± 25 ms (263 ± 29 bpm), and the blood pressure was 121 ± 14-mm-Hg systolic and 81 ± 11-mm-Hg diastolic, with no significant changes in these average measures during or after treatment. The ECG and blood pressure traces for one rat are shown in Figure 1. For this rat, the first exposure minute was triggered at R + RR/6 and the last at R + 0RR/6. The PCs and blood pressure trace show a progression of changes as the trigger point was changed from the R wave to the incrementally later times. The blood pressure trace showed that end of systole, which is marked by the brief deflection in slope caused by the closing of the aortic valve [19], occurred at approximately R + 2RR/6. Triggering at the R wave (end diastole) produced no discernable change in the signals. Triggering at R + 1RR/6 (early systole) or R + 2RR/6 (end systole) often yielded a PC in the ECG and a prolonged decrease in blood pressure during the compensatory pause before the next R wave. However, triggering during diastole at R + 3RR/6 or R + 4RR/6, which gave a PC in the ECG, elicited a premature pulse in the blood pressure signal. Triggering at R + 5RR/6 (late diastole) essentially stimulated the next heart beat to occur slightly earlier, often including the P-wave in the PC.

**Figure 2:**
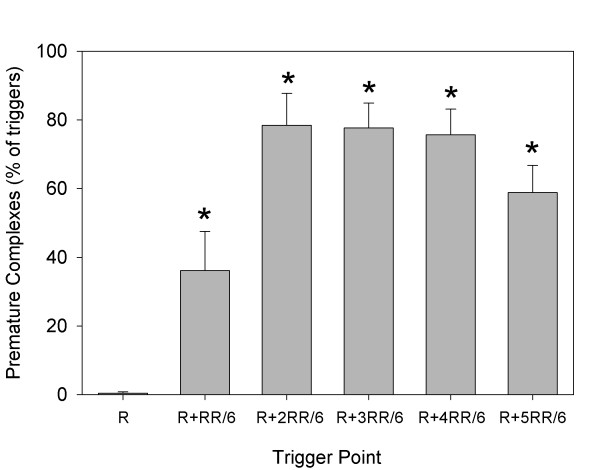
**A plot of the percentage of the triggered pulsed-ultrasound exposures, which elicited premature complexes in the ECG.** At the R wave, any electrical activity, which might have been generated by the exposure, was masked by the normal complex of the ECG, and all the other results were statistically significantly greater (asterisks) than at this time point.

**Figure 3:**
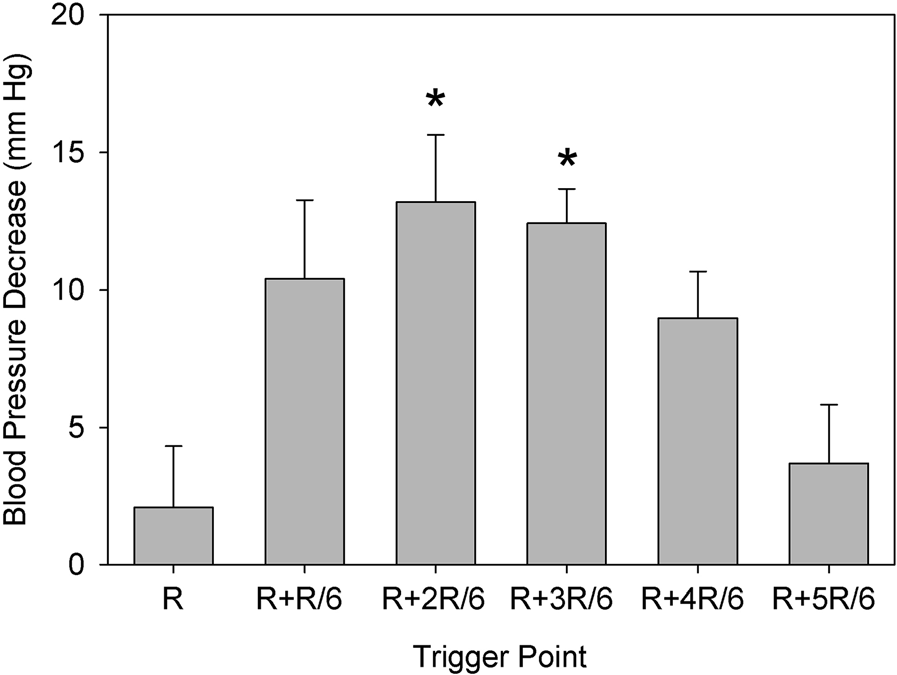
**A plot of the decreases in the end-diastolic blood pressure caused by the compensatory pauses.** The pauses followed premature complexes induced by the triggered pulsed-ultrasound exposures for the different trigger time points. The decreases for both R + 2RR/6 and R + 3RR/6 triggering were significantly greater (asterisks) than that for triggering at the R wave.

**Figure 4:**
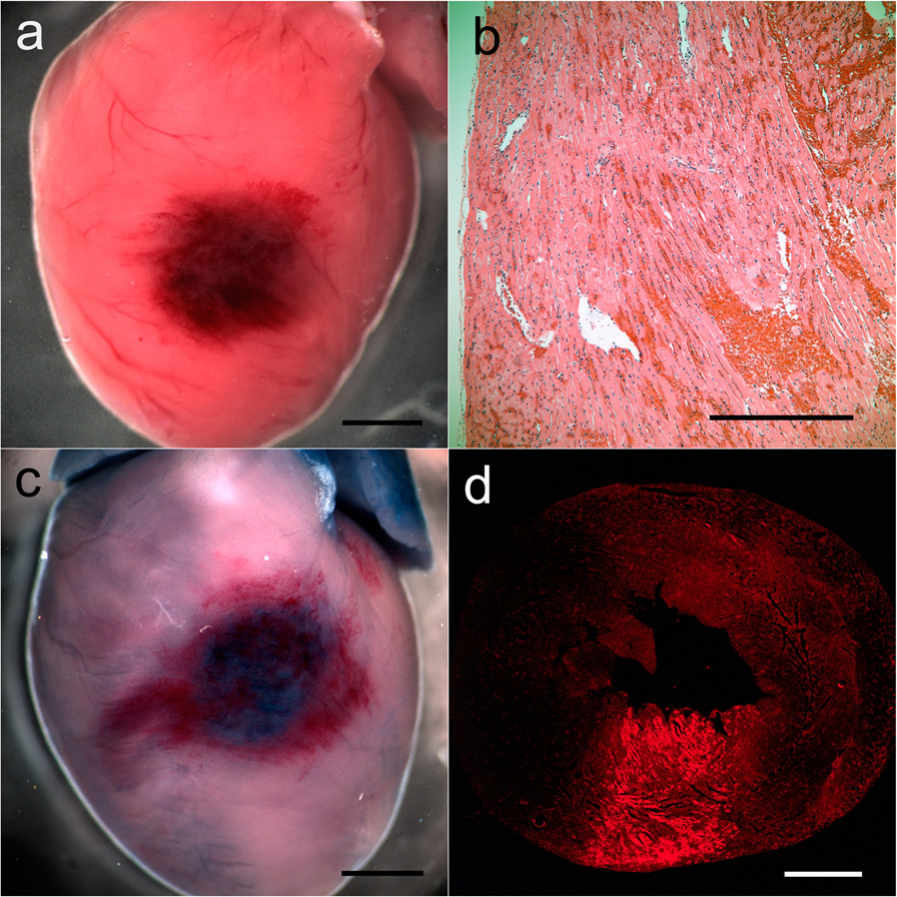
**Photomicrographs of the effects of the treatment on the heart.** The beam entry for this heart from part 1 of the study is evidenced by a red marking (**a**, scale bar 2 mm), which was due to numerous capillary hemorrhages within the myocardium (**b**, scale bar 0.5 mm). For a heart from part 2, a similar mark appears on the heart except for blue staining of injured cardiomyocytes (**c**, scale bar 2 mm), which are shown in a frozen section by red fluorescence (**d**, scale bar 2 mm).

The trends in the percentage of pulse triggers that resulted in PCs are shown in Figure 2. This percentage was significantly greater (repeated-measures ANOVA) than the very low value for triggering at the R wave (2 PCs in one rat, or 0.4% ± 1.0%) for all the other trigger points. The %PCs for R + 2RR/6, R + 3RR/6, and R + 4RR/6 were also all greater than that for R + 1RR/6 (*P* < 0.001). The blood pressure nadir at end of the compensatory pause (delayed end of diastole) also varied with trigger points, and the difference (pressure decline) between the pressure at the end of diastole before and after a PC at different trigger points is shown in Figure 3. These results were obtained by sampling six detailed measurements of heart cycles for each rat at each trigger point. The decline was as large as 13.2 ± 6.0 mm Hg for triggering at R + 2RR/6, and declines for both R + 2RR/6 and R + 3RR/6 triggering were significantly greater than that for triggering at the R wave (repeated-measures ANOVA, *P* < 0.005). One rat in the R + 1RR/6 group had PCs but no compensatory pause, which gave approximately zero blood pressure decrease for that rat, and one rat had no data due to a lack of PCs. The hearts from part 1, which were removed soon after treatment and did not contain Evans blue, showed a red lesion spot corresponding to the ultrasound focal zone entry (Figure 4a). This lesion was the result of numerous capillary hemorrhages within the tissue as shown histologically in Figure 4b.

### Part 2 results

**Figure 5:**
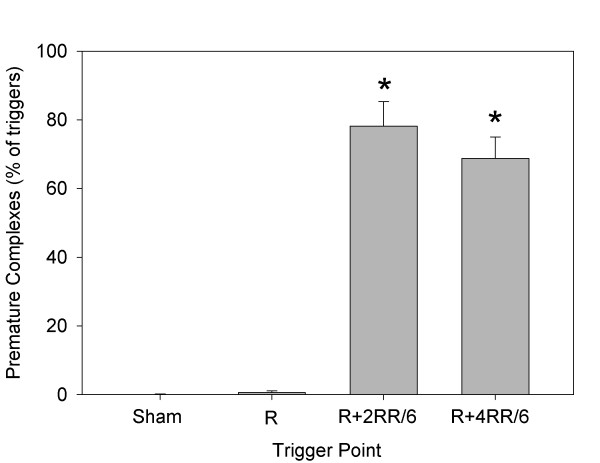
**The percentage of treatment pulse triggers resulting in premature complexes in the ECG for part 2 of the study.** The triggers at R + 2RR/6 and R + 4RR/6 produced significantly (asterisks) more premature complexes than triggering at the R wave or for sham exposure.

**Figure 6:**
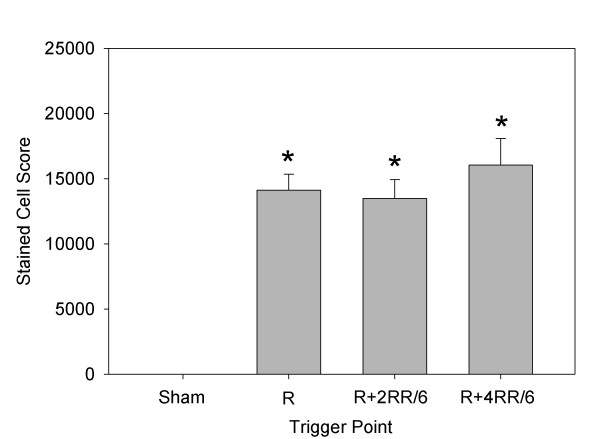
**The scores from frozen sections for cardiomyocytes stained by Evans blue.** The scores for all three trigger points were about the same and significantly greater (asterisks) than the very low scores in shams.

**Figure 7:**
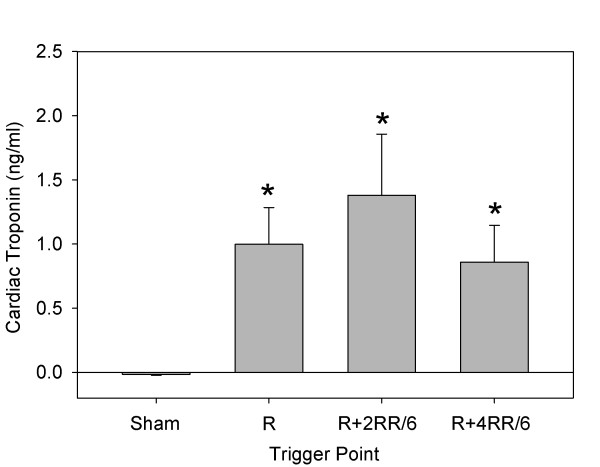
**The measurements of troponin I in plasma samples taken a day after treatment.** The results for all three trigger points were about the same and significantly greater (asterisks) than the very low scores in shams.

In part 2, the relative response was determined for triggering at the R wave, R + 2RR/6, or R + 4RR/6. The mean RR interval for rats in part 2 was 217 ± 13 ms (278 ± 17 bpm) to start and 247 ± 78 ms (255 ± 48 bpm) the next day. During treatment, the occurrence of PCs was significant (*P* < 0.001) for triggering at R + 2RR/6 or R + 4RR/6, as shown in Figure 5. A heart treated in part 2 with the R + 2RR/6 trigger point, which was subjected to overnight Evans blue staining and removed the next day, is shown in Figure 4c. The lesion area visible on the surface is similar to that shown in Figure 4a, except that the hemorrhage marking is reduced and the central region is distinctly blue. The blue region was primarily due to the presence of stained cardiomyocytes, which were scored in frozen sections under fluorescence microcopy. A fluorescence image of a frozen section at the approximate middle of the focal damage zone for this heart is shown in Figure 4d. The blue-stained cardiomyocytes in scattered microlesions appear fluorescent red, while the normal heart tissue appears darker with only background fluorescence in this 3-color image. The stained cell scores are compared in Figure 6. The scores for the treated rats were all significantly different from the shams (*P* < 0.001), but the scores were not significantly different from each other. The troponin results are compared in Figure 7. The results for the treated rats were significantly different from shams (*P* < 0.05), but there was no significant difference between the treated groups. These results for stained cell scoring and troponin both indicate that the different trigger time points produce about the same treatment impact.

## Discussion

The timing of the therapy pulses relative to the R wave can be readily varied for MCET. In this study, the influence of the trigger time point was investigated with regard to both the physiological cardiac response and the microlesion production. In the first part of the study, rats were treated for 1 min at each of six different time points in the ECG with blood pressure monitoring during treatment. This experiment revealed large differences in the functional response of the heart in terms of the ECG and blood pressure signals for different trigger time points. To evaluate the impact of the ultrasound treatment on cardiomyocyte injury, three different time points were investigated: the R wave (end diastole), R wave plus one third of the R-to-R interval (2RR/6, end systole), and R wave plus 4RR/6 (mid-diastole). The results showed no significant differences in the stained cell scores or plasma troponin measurements for these three trigger time points.

The ECG signal showed many premature complexes for trigger points other than at the R wave, see Figure 2. The blood pressure trace helped to identify key timing reference points, such as the closing of the aortic valve at end of systole, which occurred at about R + 2RR/6. The time points correspond to events in the heart cycle, which is shown in a Wiggers diagram [19]. For triggers at the R wave, there was essentially no perturbation in heart function shown by the ECG or blood pressure signals. For R + 1RR/6 and R + 2RR/6, PCs were observed but no blood pressure pulse was generated, because the heart was already in contraction with the aortic valve open. However, the blood pressure signal revealed premature pulses generated after PCs for triggering during diastole at R + 3RR/6, R + 4RR/6, and R + 5RR/6, as shown in Figure 1. These blood pulses must have required the mitral valve to re-close and the aortic valve to re-open. The compensatory pauses resulted in significant decreases in diastolic blood pressure of up to 15% at the end of diastole (Figure 3). The trigger timing at R + 5RR/6 produced minimal disturbance of the heart cycle but must have interacted with the atrial contraction occurring with the P-wave.

The optimum trigger time point appears to be at end of systole (R + 2RR/6). This setting yields clearly identifiable PCs, which are an indicator of microlesion production [14] and provide real-time feedback on treatment efficacy. The hypothesis that treatments occurring at end systole might be less effective, because of decreased myocardial vessel blood volume, was not supported (see Figures 6 and 7). Although this timing results in a prolonged compensatory pause, which reduces end-diastolic blood pressure for that cycle and may be felt by patients who are awake, the PCs appear to be pulseless electrical signals. If the trigger timing is set in diastole, then a pulse is generated by premature ventricular contractions and stressful valve actuation might occur. This finding further refines MCET procedures, which also may be applicable to other cardiac therapy methods, such as drug, gene, or stem cell delivery. Further research for development of better microlesion scoring is in progress [20]. In addition, improved real-time monitoring to control and define the actual tissue reduction created will be needed for safe and effective therapeutic application.

## Conclusion

MCET allows the opportunity to adjust the timing of therapy pulse-bursts within the cardiac cycle. The end of systole (R + RR/3) was identified as an optimal trigger time point which produced identifiable ECG complexes and substantial cardiomyocyte injury but minimal cardiac functional disruption during treatment.

## Competing interests

The authors declare that they have no competing interests.

## Authors’ contributions

The experimental design, data acquisition, and interpretation were performed by all authors. An initial draft manuscript was prepared by DLM and revised by GEO and ODK. All authors read and approved the final manuscript.
